# Does opt-out legislation crowd out living organ donations? A cross-country study

**DOI:** 10.1007/s10198-025-01825-z

**Published:** 2025-10-14

**Authors:** Stefan Felder, Richard Abbasi

**Affiliations:** Faculty of Business and Economics, University of Basel, Peter Merian-Weg 6, Basel, 4002 Switzerland

**Keywords:** Organ donations, Presumed consent, Crowding out of living donations, I18, K32, D82

## Abstract

To address the shortage of donor organs, some countries have adopted opt-out legislation, whereby consent for post-mortem donation is presumed unless individuals explicitly object prior to death. However, an increase in post-mortem organ donations may reduce the willingness of relatives to make living donations. We test this hypothesis using data from 26 countries over a 21-year period. Using fatal injuries as an instrument for cadaveric kidney supply, we find a significant effect on living donation rates; 50% of the higher living donation rate observed in opt-in countries can be attributed to a lower supply of cadaveric organ transplants.

## 1 Introduction

The predominant legal framework divides organ donation policy into two defaults: opt-in and opt-out. Under opt-in, only individuals who gave consent while alive can become postmortem donors. Opt-out presumes consent unless expressly denied, thereby expanding the potential donor pool to include those who were willing but never registered [1].

The persistent shortage of donor organs in all countries results in long waiting lists. For instance, in the US in 2019, 113,000 patients were on the waiting list. Depending on the organ type, average wait times ranged from 213 to 370 days [2]. As a result, 4% of individuals on the waiting list died before a graft became available. In 2022, Switzerland had 1,442 candidates, 83 (5.8%) of whom died that same year [3]. Efforts to increase donation rates have prompted several countries – including Wales, England, the Netherlands, and, most recently, Switzerland – to shift from opt-in to opt-out legislation.

Empirical evidence on the effectiveness of opt-out systems remains mixed. While some studies associate optout systems with higher transplant rates (see [4, 5] for systematic reviews [6]), the cross-country comparison by Arshad et al. [7] shows no such advantage. Shepherd et al. [8] identified clear differences across legislative defaults and emphasise the importance of distinguishing between living and deceased donors. Remarkably, the number of living kidney transplants is higher in opt-in than in opt-out countries [9] and makes up about one-third of total kidney donations. Our dataset covering 26 countries over 21 years reveals a clear pattern: cadaveric donation rates are 6.86 per million population (pmp) higher in opt-out countries while living donation rates are 6.81 pmp higher in opt-in countries (Table 1).

**Table 1:** Average organ transplant rates (per million population) and the predominant procurement regime (2002–2022). See Table 7 in the Appendix on each countries legislative status and relevant legal text

	Legislation		Difference
	Opt-out	Opt-in	
**Total**	**62.41**	**62.36**	**0.05**
Kidney	39.30	38.37	0.93
Liver	13.21	11.45	1.76
**Total cadaveric organs**	**56.28**	**49.42**	**6.86**
Kidney	33.58	26.02	7.56
Liver	12.80	10.86	1.94
Heart	4.80	4.23	0.57
Lung	3.60	4.25	−0.66
Pancreas	1.51	1.35	0.16
**Total living donations**	**6.12**	**12.94**	**−6.81**
Kidney	5.71	12.35	−6.63
Liver	0.41	0.59	−0.18
Number of countries	16	10	26
Number of observations	339	207	546

Opt-out: Austria, Belgium, Czech Republic, Estonia, Finland, France, Hungary, Italy, Latvia, Netherlands (2020–2022), Norway, Poland, Portugal, Slovakia, Slovenia, Spain, Sweden

Opt-in: Australia, Canada, Denmark, Germany, Ireland, Lithuania, Netherlands (2002–2019), New Zealand, Switzerland, US

Sources: [10, 11]

Güntürkün et al. [12] identified a supply–demand crowding-out effect on living donations. They report a substantial spill-over effect of presumed consent on living donations, thereby suggesting that switching from opt-in to opt-out may result in a negative net effect of − 0.77.^1^ This paper refines the measurement of the crowding out mechanism. Shepherd et al. [8] used an instrumental variable (IV) approach when comparing living and deceased donors. Their instruments – civic engagement indicators such as volunteering and charitable giving – reflected the likelihood that a country would adopt opt-out legislation. We extend that inquiry by assessing how a change in cadaveric organ availability affects relatives’ willingness to donate. Our IV design estimates the decline in living donations associated with each additional cadaveric transplant, thereby clarifying the substitution effect. This focus addresses a gap in the literature by showing that legal defaults influence both the supply of deceased organs and the behaviour of prospective living donors.

Living donations pertain^2^ to kidneys and livers. Liver donation rates are low with only small differences between opt-in and opt-out countries. Kidney donation rates are substantially higher with cadaveric rates 7.56 pmp higher in opt-out countries. An expanded cadaveric organ pool may reduce the need for living donations by increasing the likelihood that a patient receives an organ without relying on a relative. If so, the lower average rate of living donations in opt-out countries (–6.81 pmp) might reflect a crowding-out effect resulting from higher cadaveric organ supply. This paper tests this conjecture.

The partial removal of the liver from a living donor is a complicated medical intervention. The number of liver donations is low and often non-existent in many countries. For this reason, we do not consider liver transplants and restrict our analysis to kidney transplants.

The remainder is organised as follows. In Section 2, we model the effect of opt-out legislation on cadaveric donation rates and examine the relationship between the supply of living and cadaveric donations. The data are presented in Section 3, the empirical results in Section 4, and the discussion in Section 5.

## The effects of opt-out legislation

At first glance, it is unclear whether the organ-procurement default affects the equilibrium transplant rate, especially for living donations, which typically involve an intimate agreement between relatives^3^. However, even for post-mortem organ donations, the ability to freely state one’s preference may reduce the relevance of the legal default, whether opt-in or opt-out. Nevertheless, declaring a preference involves a cost^4^, and some individuals refrain from expressing their support for or opposition to the existing regime. In the German opt‑in system, a representative survey found that 17% of respondents had a preference for organ donation but had not formally expressed it [13]. Likewise, an earlier US study found that 12.8% of the population held weak preferences for donation [14].

### Post-mortem donations

Abadie and Gay [15] explained why, when declarations are costly, opt-out legislation can be expected to yield more donations than opt-in legislation. Assume an individual’s utility gain from an organ donation is $\:{v=u}_{D}-{u}_{N}$, where $\:{u}_{D}$ and $\:{u}_{N}$ represent the respective utilities from donation and non-donation. Individuals of Type *D* prefer a donation ($\:v>0$), whereas those of Type *N* do not ($\:v<0$).

**Fig. 1:**
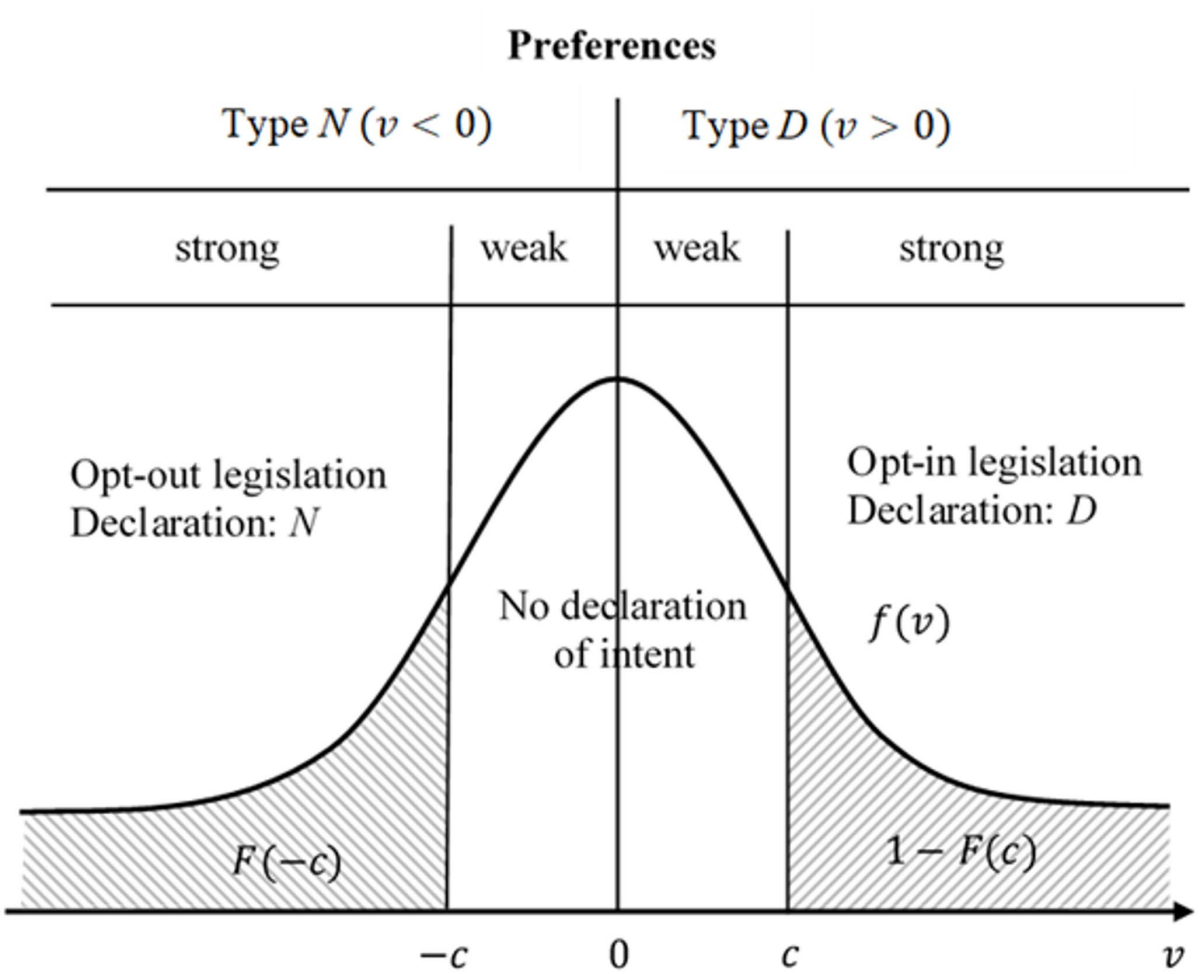
Rate of donations and the predominant donation regime

The decision to reveal preferences is illustrated in Fig. 1, where $\:f\left(v\right)$ represents the density and $\:F\left(v\right)$ the distribution of *v*. Given the declaration cost *c*, under opt-in legislation, a portion of the population $\:1-F\left(c\right)$ has a strong preference for *D* and will thus declare their willingness to donate. A portion equal to $\:F\left(c\right)-F\left(0\right)$ will not despite having a weak preference for *D*. Under opt-out legislation, the portion $\:F(-c)$ of the population will reveal their type *N* while a portion of $\:F\left(0\right)-F(-c)$ with a weak preference for *N* will not. If the legal default is followed, the donation rate is higher under opt-out legislation because $\:F\left(c\right)-F\left(-c\right)>0$. Even in the absence of a declared preference by the deceased, families generally do not override the default legal regime^5^. As psychological findings suggest (e.g [16]), individuals tend to prefer inaction over risking a wrong choice when information is incomplete. This reasoning leads to the following proposition.

- Proposition 1: Opt-out countries have a higher average cadaveric organ donation rate than opt-in countries.

### Living donations

Domínguez-Gil and Pascual [17] explored strategies to increase the number of living organ donations in Spain. Although cadaveric donation rates are high, the country continues to face a shortage of organ donors. The authors noted that the number of living donations is relatively low, possibly because of the high rate of cadaveric organ supply.

Consider the decision-making process of an individual whose family member is awaiting a kidney transplant. Let *u* represent the recipient’s utility gain from receiving an organ and *p* the probability of obtaining one. Let *l* represent the individual’s utility loss from donating an organ and α ∈ [0,1] their degree of altruism. The individual will be willing to donate if the utility derived from donating an organ exceeds the utility they obtains from their relative receiving a cadaveric donation:

1 $$\alpha\:u-l\ge\:p\times\:\alpha\:u$$

Willingness to make a living donation clearly requires some degree of altruism on the part of the potential donor (i.e. $\alpha>0$). Moreover, a higher probability of obtaining a cadaveric organ mitigates the individual’s willingness to make a living donation. If we let *d* denote the demand for and *s* the supply of cadaveric organs at a given time, we have $\:p=s/d$. This leads us to hypothesise that a larger supply of cadaveric organs decreases the supply of living donations.

In practice, several agents influence an individual’s decision to donate an organ. We model the resulting supply of living donations as a sequential three-stage decision involving the doctor, the organ recipient, and the donor.

The patient’s doctor assesses the need for an organ transplant and decides whether to place the patient on the cadaveric kidney waiting list. When the rate of cadaveric kidney transplant increases, a patient’s prospect of receiving a kidney in time improves. Given the emotional strain and time commitment associated with a living donation, doctors might be reluctant to initiate corresponding discussions, especially when cadaveric organ supply is high [18].

Among the three agents, potential donors bear the greatest costs. Once compatibility is established, they must weigh surgical risks and potential long-term health effects against the psychological burden of refusing to donate. This can lead to considerable emotional distress or guilt. Additionally, the organ recipient might refuse a living kidney donation in order to avoid imposing such costs on a close donor [19]. Improved access to cadaveric organs reduces the urgency for living donations, thereby alleviating some of the psychological burden on potential donors. However, this reduced urgency may also result in fewer donors coming forward, even among those who are compatible and willing to consider donation under less favourable circumstances.

This framework predicts that countries with higher cadaveric donation rates have lower living donation rates. If Proposition 1 holds (i.e. opt-out countries have higher cadaveric transplant rates), we can expect lower living donation rates in opt-out countries than in opt-in countries. Furthermore, Eq. 1 implies that changes in cadaveric transplant rates more strongly affect the probability $\:p$ of receiving an organ in countries with low cadaveric transplant rates. This suggests that substitution effects are more pronounced in opt-in countries. The following propositions summarise these claims.

- Proposition 2: Countries with higher cadaveric donation rates tend to have lower living donation rates.
- Proposition 3: The substitution effect from changes in cadaveric organ supply is stronger in countries with a lower cadaveric donation rate.

## Data

The sample includes countries with sufficiently high transplant activity, where excess demand reflects a shortage of donors rather than limitations in medical infrastructure. Following Abadie and Gay [15], we include only countries with an overall transplant activity of at least 20 pmp and a GDP per capita exceeding USD 18,000 (as of 2022) [20]. Abadie and Gay [15] argued for the inclusion of only western Christian countries in order to ensure comparable socio-economic factors. For instance, Turkey is excluded despite its relatively high per capita GDP. The UK is also excluded because of differing organ donation legislation across its nations [21] as well as the lack of disaggregated data. The resulting sample includes 26 countries (10 with an opt-in and 16 with an opt-out regime) with annual data spanning from 2002 to 2022. The Netherlands are included in both groups because the country changed its legislation from opt-in to opt-out in 2020.

Data on cadaveric and living organ transplants were sourced from the International Registry in Organ Donation and Transplant [11] and the Global Observatory on Donation Transplant [10]. The statistics include the number of kidney, liver, lung, and heart transplants as well as aggregate numbers for living and post-mortem donations. Specifically, the data include the number of kidney and liver transplants from both living and post-mortem donors.

Our analysis focuses on the number of transplanted organs rather than the number of donors. A living donor typically provides only one organ, whereas a post-mortem donor can provide multiple organs to several recipients^6^. Examining transplant rates thus allows for greater comparability. Population data are obtained from the United Nations Population Fund report [22], and information on the legal status of organ donation in each country is sourced from Shepherd et al. [8].

Previous research has shown that a range of covariates greatly influence a country’s organ transplant rate. These typically include economic, legal, social, and mortality-related indicators, which are used as control variables. Similar to the approach of Shepherd et al. [8], we use these covariates to explain cross-country differences in transplant rates rather than variations in these rates over time within individual countries. This approach allows for a distinct examination of standard errors, thereby yielding more robust results.

Both GDP and health expenditures per capita (both in PPP USD) are included as economic and health-related indicators with data sourced from the International Monetary Fund [20]. Previous research has also shown that organ transplant rates differ between common-law and civil-law countries. We account for this using a dummy variable set to 1 for common-law systems and 0 for civil-law systems.

Building on evidence provided by Abadie and Gay [15] and Gimbel et al. [23], which suggests that countries with a strong Catholic presence tend to have higher cadaveric transplant rates, the proportion of self-identified Catholics is included as an additional covariate. The data for this variable are obtained from the World Christian Database [24], an online resource providing demographic statistics on global religions. Following Shepherd et al. [8], we categorise this variable into groups rather than treating it as continuous.

The rate of fatal injuries from external sources such as traffic accidents and violence is included as a mortality indicator (sourced from [25]). According to the Organ Procurement and Transplant Network [26], 15% of organ donations in the US originate from traffic fatalities, thus highlighting the relevance of this measure.

## Empirical results

**Fig. 2:**
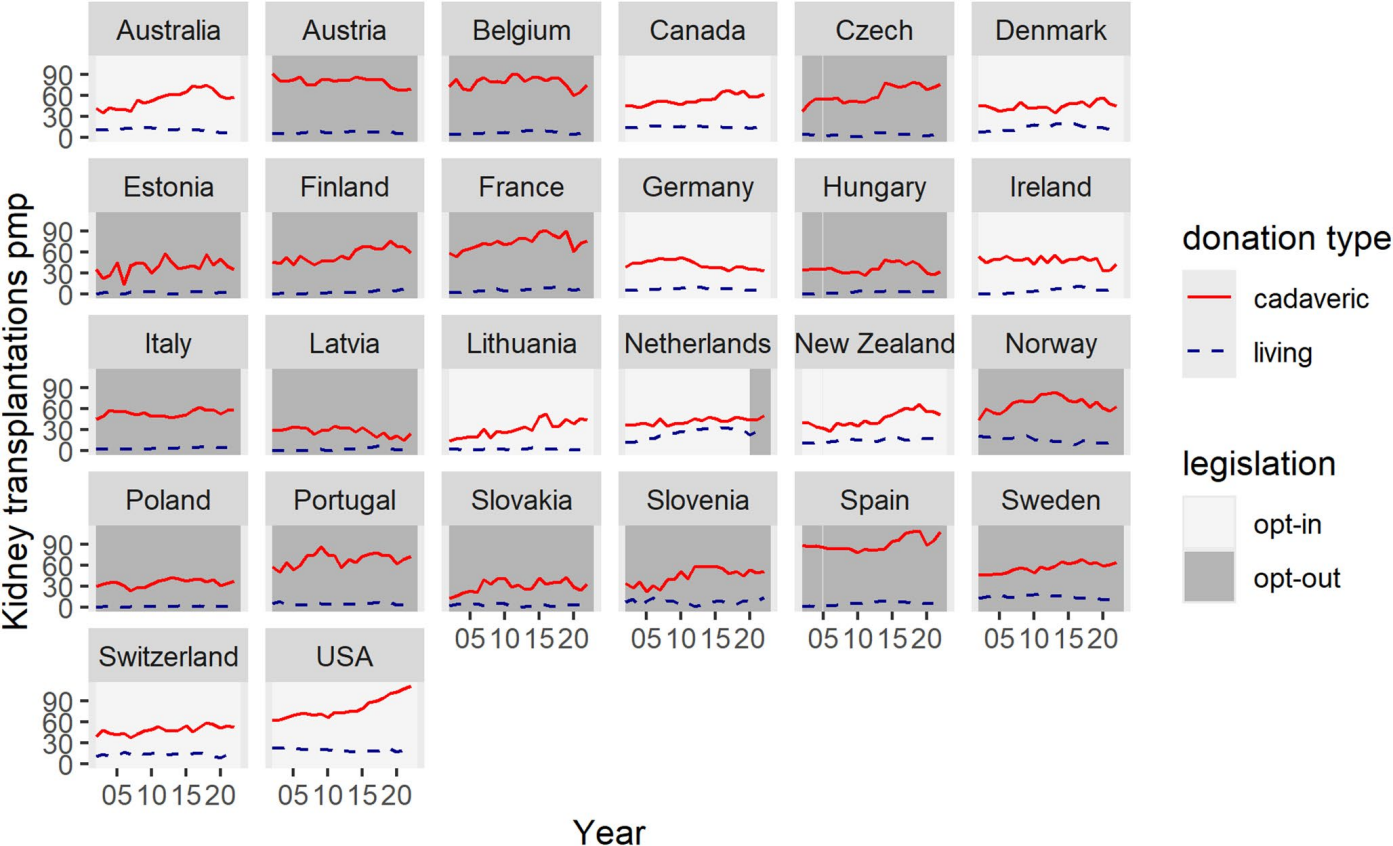
Organ transplants per million population 2002–2022, 26 countries

As depicted in Fig. 2, organ transplant rates vary considerably across countries. Although the general trend shows an increase in cadaveric rates over time (indicated by red lines), Germany, Ireland, Austria, Belgium, and Latvia deviate from this pattern. The average initial transplant rate is 44.82 pmp (39.94 for opt-in and 47.86 for opt-out). The mean annual rate of change is 0.68 pmp. However, the data reveal a decline in transplant rates for countries such as Spain and Ireland during the years 2020 and 2021; this is likely attributable to the COVID-19 pandemic. Apart from these anomalies, the relationship between time and transplant rates appears to be linear throughout the observation period with minimal year-to-year fluctuations.

The US shows a relatively strong increase over the period (an average of 2.33 pmp per year), whereas Germany shows the most pronounced decline (an average of − 0.68 pmp per year). Moreover, the slopes of the time series for opt-in (represented by a white background) and opt-out (grey background) countries do not show significant differences (0.56 pmp and 0.85 pmp, respectively).

When examining the time series of living organ donations (represented by dashed blue lines), cross-country heterogeneity appears less pronounced, and within-country fluctuations over time are smaller. The mean intercept is 10.14 pmp for opt-in countries and 4.83 pmp for opt-out countries. Overall, living donation rates are lower than cadaveric donation rates. Nevertheless, 89% of the variation is attributable to differences between countries. There is a weak positive trend with an average slope of 0.10 pmp for opt-in countries and 0.12 pmp for opt-out countries. Some countries also experienced a decline in living donations during the COVID-19 pandemic. A comparison of the intercepts indicates that opt-in countries have higher living transplant rates.

In addition to the information presented in Tables 1 and 2 shows further differences between the two groups of countries. Notably, all five common-law countries in the sample have opt-in legislation, reflecting a preference for informed consent in these jurisdictions. The rate of traffic fatalities does not differ much between opt-out and opt-in countries. On average, population size is three times larger in opt-in countries. This is largely due to the inclusion of the two most populous countries in this group. Additionally, the groups differ economically with respect to health spending and GDP per capita.

**Table 2:** Descriptive statistics of the covariates, 2002–2022

	Legislation				
	Opt-out	Opt-in	Difference	s.e.	*p*-value
Cadaveric transplants (pmp)	56.28	46.97	9.31	6.38	0.15
Living donations (pmp)	6.12	12.94	−6.82	2.28	0.00
Population (in millions)	17.92	50.21	−32.29	30.30	0.29
GDP per capita (in thousands of USD)	31.74	49.54	−17.80	7.66	0.02
Health expenditure per capita (in thousands of USD)	2.90	5.02	−2.13	0.83	0.01
Mortality from external causes (pmp)	396.40	339.16	57.24	71.01	0.42
	N	N			
Proportion of Catholics ≤ 50% (> 50%)	6 (10)	8 (2)			
LawCivil (Common)	16 (0)	5 (5)			

Notes: This table presents descriptive statistics comparing opt-in and opt-out countries in terms of organ donation rates, demographic characteristics, economic indicators, and legal systems. Reported *p*-values are derived from Student’s t-tests with standard errors clustered at the country level. Mortality from external causes is defined according to the OECD ICD-10 classification (Chapter XX)

Sources: [10, 11, 20, 22, 24, 25]

The proportion of Catholics is nearly collinear with the procurement rule: while 10 of the 16 opt-out countries have a Catholic population exceeding 50%, only two of the 10 opt-in countries cross this threshold. This pattern may suggest that a country’s choice of organ donation legislation is influenced by religious beliefs, thereby calling into question the exogeneity of the legal framework. Such collinearity suggests that religious composition may confound the estimated effect of legislation on organ donation rates and should be carefully accounted for in any causal analysis.

With this sample, a fixed-effects model is not suitable for quantifying the effect of consent legislation because only the Netherlands changed its default within the sample period. The estimate would heavily rely on this single observation and could lead to false conclusions, particularly because the legislative change coincided with the COVID-19 pandemic. Removing this observation from the sample would eliminate the variable of interest because it would be absorbed by the country fixed effects.

Our analysis indicates that 86% of the observed variance is due to differences between countries^7^ while the remaining 14% is associated with temporal changes. This highlights the importance of considering the hierarchical structure in our analysis. To address between-country differences in organ transplant rates and within-country temporal variations, we conducted multi-level regression analyses to assess the effect of consent laws on cadaveric and living donation rates.

At the first level, the transplant rate $\:{Y}_{i,t}$ trajectories for each country $\:i$ are regressed on a linear time trend and a COVID-19 time dummy variable, both included in the matrix $\:{T}_{i}$. Therefore, for each country a regression of the form

2 $${Y}_{i,t}={\beta\:}_{0,i}+{\beta\:}_{1,i}{T}_{i,t}+{\varepsilon\:}_{i,t}$$

is estimated. Here, $\:{\beta\:}_{0,i}$ represents the country-specific intercept, $\:{\beta\:}_{1,i}$ the country-specific slope and COVID-19 effect, and $\:{\varepsilon\:}_{i,t}$ the errors.

The second level of the regression incorporates covariates $\:{X}_{1}$ that predict the random intercepts $\:{\beta\:}_{0,i}$ and slopes $\:{\beta\:}_{1,i}$ derived from the first-level regression. This is modeled by a system of two equations:

3 $${\beta\:}_{0,i}={\beta\:}_{00}+{\beta\:}_{01}{X}_{1}+{\varepsilon\:}_{0,i}$$

4 $${\beta\:}_{1,i}={\beta\:}_{10}+{\beta\:}_{11}{X}_{1}+{\varepsilon\:}_{1,i},$$

where $\:{\beta\:}_{00}$ is the fixed effect for the intercept, $\:{\beta\:}_{10}$ the fixed effect for the slope $\:T$, $\:{\beta\:}_{01}$ the fixed effect of $\:{X}_{1}$ for the intercept, and $\:{\beta\:}_{11}$ the fixed effect of $\:{X}_{1}$ for the slope $\:T$ [27]. Table 3 presents the second-level regression results of our analysis, incorporating covariates such as predominant legislation, religion, and common-law dummy variables along with the logarithm of the average health expenditures per capita.

**Table 3:** Explaining organ donation rates (2002–2022)

	Total			Kidney	
	Cadaveric		Cadaveric	Living	Difference
	(1)	(2)	(3)	(4)	(5)
Opt-out consent	9.267** (4.256)	9.646^**^ (3.851)	6.884^***^ (2.432)	−4.974^***^ (1.157)	11.827^***^ (2.791)
Log per capita health expenditure	17.798*** (3.430)	17.794*** (3.284)	4.541^**^ (1.938)	3.306^***^ (1.197)	1.140 (2.318)
Catholic country	11.287** (4.937)	11.331** (4.813)	4.048 (2.836)	−2.999^*^ (1.758)	8.576^**^ (3.544)
Common-law	−0.640 (6.615)				
Constant	37.019*** (4.422)	36.802*** (3.600)	22.750^***^ (2.165)	11.915^***^ (1.280)	11.163^***^ (2.624)
Observations	546	546	546	546	546
Level 1 *R*^2^	0.896	0.895	0.797	0.944	0.886
Level 2 *R*^2^	0.438	0.429	0.197	0.478	0.358
RMSE	5.563	5.565	3.891	1.614	4.290

Notes: ^*^p < 0.1; ^**^p < 0.05; ^***^p < 0.01. Reported coefficients refer to the variables used in the level 2 regression. Outcome variables are the number of transplants with organs from deceased donors per million persons (pmp) in Columns 1 and 2, the transplant rate of cadaveric kidneys pmp in Column 3, and the transplant rate of living kidney donors pmp in Column 4. The clustered standard errors are in parentheses. The variable “Log per capita health expenditure” represents the logarithm of the country-averaged per capita health expenses from 2002 to 2022. A country is classified as Catholic if over 50% of its population identifies as Catholic. We conducted the analysis in *R* with the lme4 package

Models 1 and 2 provide estimates for the total cadaveric transplant rates. When controlling for per capita health expenditure and religion, the difference in transplant rates associated with consent legislation becomes more pronounced than suggested by the summary statistics: opt-out consent regimes are linked to more than nine additional transplants pmp per year, thereby supporting the validity of Proposition 1. The significant coefficient for per capita health expenditure suggests that a country’s health infrastructure plays an important role in determining the number of cadaveric organ donations. Additionally, countries with a high proportion of Catholics are associated with more cadaveric transplants. Given the *t*-values of the coefficients in Model 1, it is evident that the common-law variable is not significantly associated with variation in transplant rates.

Relatively high $\:{R}^{2}$ values in the level 1 regressions indicate that the specific transplant rates for each country follow a linear time trend. In contrast, the modest $\:{R}^{2}$ values of the level 2 regressions reveal that country-specific indicators can explain less than half of the variation between countries. A residual mean squared error (RMSE) of 5.6 represents the magnitude of the unexplained variation. Relative to the average cadaveric transplant rates, the standard error remains relatively low at 10%.

Models 3 and 4 separately analyse cadaveric and living kidney transplant rates. The comparison between these two models confirms the opposite effects of opt-out legislation on cadaveric and living donations. Although consent legislation does not regulate living donations, the estimation shows a significant negative effect on living kidney transplants. This may result from the indirect effect of consent legislation on living donations Models 3 and 4 differ in their constants, thereby precluding a direct comparison of their outcomes. To address this, Model 5 specifically analyses the differences between cadaveric and living transplant rates. The results demonstrate that Catholic countries exhibit a significantly larger gap between cadaveric and living donation rates, further clarifying the relationship between religious composition and organ donation patterns. This relationship may be driven by the preference for an opt-out regime in predominantly Catholic countries, thereby suggesting endogeneity in the legislative variable. Shepherd et al. [8] made a related argument regarding the role of common law. However, in the IV analysis, we can disregard this by using fixed effects models, which account for both legislation and religion within the fixed country effects.

The lower goodness of fit in the third model specification indicates that kidney transplants account for most of the unexplained variability in cadaveric transplant rates, largely because several countries maintain low but stable living donation activity. However, transplant activity fluctuates less for living than for cadaveric donations (Fig. 2). The lower RMSE for living donations can be attributed to the smaller scale of the outcome variables.

The intended model we aim to estimate is

5 $${Y}_{i,t}^{liv}={\alpha\:}_{i}+\beta\:*{Y}_{i,t}^{cad}+\gamma\:*{X}_{i,t}+{\varepsilon\:}_{i,t}\:,$$

where $\:{Y}_{i,t}^{liv}$ ($\:{Y}_{i,t}^{cad}$) represents the kidney living (cadaveric) transplant rate pmp for country $\:i$ in year $\:t$. The country fixed effects are given by $\:{\alpha\:}_{i}$, $\:\beta\:$ represents the coefficient for $\:{Y}_{i,t}^{cad}$, and $\:\gamma\:$ is a vector of coefficients for covariates such as “log per capita health expenditure,” “Year,” and “COVID-19,” all included in the matrix $\:{X}_{i,t}$. The errors are represented by $\:{\varepsilon\:}_{i,t}$.

Opt-out legislation has been shown to increase cadaveric organ transplant rates; this may, in turn, reduce incentives for living donations. Our estimation supports Proposition 2, indicating that countries with high cadaveric organ transplant activity tend to have fewer living donations (see Table 4, Model 1). However, the supply of cadaveric donations is not exogenous. Addressing the problem of endogeneity requires an instrument that influences the dependent variable solely through its effect on the endogenous regressor. Our instrument must therefore be correlated with cadaveric transplant rates and affect living donation rates only by altering the availability of cadaveric organs. The first condition can be tested by assessing the significance of the first-stage regression while the second condition must be established intuitively.

**Table 4:** Explaining living kidney donation rates (2002–2022)

	Living	Cadaveric	Living
	panel linear	first stage	IV
	(1)	(2)	(3)
Cadaveric kidney rate	−0.070^***^ (0.025)		−0.361^***^ (0.096)
Log per capita health expenditure	−0.119 (0.740)	0.343 (1.391)	0.066 (0.867)
Mortality from accidents		0.036^***^ (0.006)	
Year	0.187^***^ (0.035)	0.468^***^ (0.068)	0.278^***^ (0.049)
COVID-19	−2.446^***^ (0.458)	−4.487^***^ (0.833)	−3.586^***^ (0.637)
Observations	462	462	462
*R* ^2^	0.864	0.797	0.821
Adjusted *R*^2^	0.857	0.785	0.811
RMSE	2.507	4.632	2.880

Note: ^*^p < 0.1; ^**^p < 0.05; ^***^p < 0.01. All specifications include country fixed effects with clustered standard errors in parentheses. Column 1 presents the results of the fixed effects regression without controlling for the endogeneity of the cadaveric kidney rate (see Eq. 5). Column 2 shows the first stage regression results, whereby the cadaveric kidney transplant rate is regressed on mortality from external causes, the time trend, and the COVID-19 fixed effect. Column 3 displays the IV estimation results using the same control variables as in Column 2. We conducted the regressions in *R* with the ivreg package

Mortality rates serve as a direct indicator influencing the supply of cadaveric donations without directly affecting living donations. We hypothesise that higher mortality rates increase organ supply, thereby reducing incentives for living donations. Sheehy et al. [28] found a positive correlation between accident-related mortality and cadaveric organ transplant rates for the US. Nevertheless, some countries show decreasing mortality alongside increasing transplant rates, suggesting that additional factors such as improved road safety and shifting demographics play a role. Consequently, the variation in transplant activity across countries cannot be fully explained by mortality alone, thereby necessitating the inclusion of control variables.

Although the relationship between mortality rates and cadaveric donations is straightforward, their indirect effects on living donations cannot be entirely dismissed. One potential mechanism involves the effect of mortality rates on organ demand. High accident-related mortality rates often coincide with a greater incidence of accidents, thereby potentially increasing the demand for organ transplants. For instance, severe accidents might result in the loss of both kidneys, thereby creating the need for a transplant for a surviving individual. While such cases are plausible, they are likely rare and thus assumed to have minimal effect on overall organ demand.

Another important consideration is the potential endogeneity of mortality rates. Mortality rates vary across countries because of factors such as demographic structures and healthcare policies. To address these variations, we incorporate fixed country effects in our model. Nevertheless, policies influencing mortality rates may affect our findings. However, in the absence of observable abrupt changes or significantly shifts in mortality rates, we assume that policy-driven variations are unlikely to substantially influence the results. To test this assumption, we conducted a placebo test using kidney-related mortality as an instrument.^8^(Table 6) The placebo test yields no significant effect, thereby supporting the robustness of our model.

Building on these findings, we identify “external causes of morbidity and mortality”^9^ among individuals aged 20 to 80 years^10^ as a suitable instrument^11^. Measurement errors in the mortality data lead us to remove four countries^12^ from the sample, thereby resulting in a final analysis with 22 countries.

Unlike in the previous analysis, we no longer focus on the composition of fixed and random effects. Using a fixed-effects model, we control for country-specific heterogeneity – including cultural influences on willingness to donate – to obtain a more accurate estimate of the effect of cadaveric transplant supply on living donation rates. Specification 1 presents the results of the intended model without addressing endogeneity. The coefficient (− 0.070) indicates a negative correlation between living and cadaveric transplant rates.

In the first stage, the cadaveric transplant rate is regressed on the mortality rate along with covariates using fixed country effects. The statistically significant coefficient of the instrument (see (2) in Table 4) confirms the robustness of the first-stage estimation. A *t*-value of 48.2 rejects the null hypothesis that our instrument is weak. The first-stage regression predicts that a one-unit increase in mortality is associated with a 0.036 increase in cadaveric kidney transplants. In other words, 3.6% of deaths caused by external factors result in kidney donations.

In the second stage, living donations are regressed on the predicted values from the first stage along with covariates and fixed country effects. The Wu-Hausman test, which checks for the endogeneity of a variable, yields a *p*-value of 0.002, thereby indicating superiority of the IV regression over the model without instruments. The OLS estimates as shown in (1) are therefore biased and inconsistent despite the lower RMSE suggesting a better fit.

Comparing the $\:{R}^{2}$ and RMSE of the model with and without IV reveals that the residuals are underestimated without IV. This further indicates the endogeneity of the cadaveric transplant rates.

The substantial IV coefficient suggests that each additional cadaveric kidney reduces living donations by 0.36 (this corresponds to roughly to one fewer living donation for every three extra cadaveric donations). Opt-out consent procurement rules account for an increase in cadaveric kidney supply of 6.89 pmp (Table 2). This, in turn, reduces living donations by 2.49 pmp. This reduction also explains 50% of the 4.97 pmp lower rate of living donations observed in opt-in countries.

Because of the relatively small sample size of 22 countries, the estimates of the effect of cadaveric kidney transplant on living kidney donations might be sensitive to sample selection. This sensitivity is reflected in heterogeneous effects for different sub-samples (Appendix Table 5). When restricting the IV estimation to opt-in countries^13^, we find a significant effect of − 0.320, similar to the − 0.361 result obtained using the whole sample. This result supports Proposition 3, thereby indicating an inverse linear relationship between the probabilities of receiving a cadaveric organ and receiving an organ from a living donor. We can therefore exclude the possibility that our findings are driven mainly by opt-out countries, which exhibit greater imbalances between cadaveric and living donations.

In order to reflect changes in practice patterns and healthcare delivery, we include a dummy variable for the COVID-19 pandemic. Restricting the sample to pre-2020 yields an effect of − 0.381 (*t* = − 2.61), thereby indicating that the dummy variable appropriately accounts for pandemic-related disruptions.

## Discussion and conclusion

Research on consent legislation has predominantly focused on cadaveric donations [5, 15, 23] given that consent laws primarily govern post-mortem organ donation. However, acknowledging systematic differences between cadaveric and living donation rates, some researchers have examined the effect of presumed consent legislation on living donation rates (e.g [8]). Nevertheless, the specific relationship between cadaveric and living donation rates so far has not been examined. We use an IV approach to model this relationship.

Transplant statistics suggest a negative correlation between cadaveric and living donation rates. Countries with high cadaveric donation activity often exhibit lower living donation rates. For example, Spain has the highest cadaveric donation rates yet relatively low living donation activity [29]. A survey indicated that this might result from doctor behaviour because 83.4% of patients placed on a waiting list were not informed about living donation as a treatment option. Despite an organ shortage, most doctors considered cadaveric transplants sufficient and avoided discussions about alternatives [17]. Such clinical gatekeeping can restrict patient awareness of alternative transplant options, leading to the underutilisation of living donors despite the availability of altruistic support from relatives or friends. Conversely, countries with low cadaveric donation rates tend to have the highest living donation rates globally. This is the case in Turkey and Saudi Arabia, where the shortage of post-mortem donations is due partly to religious convictions. We argue that the legislative environment for post-mortem donation is less likely to influence the decisions of potential living donors because living donation typically arises from direct personal relationships or altruistic motivations.

The results from our IV approach indicate that an individual is more likely to donate a kidney to a family member if the likelihood of receiving an organ from a cadaveric donor is low. This relationship is demonstrated by an estimated reduction in living donations of approx. one third for each additional cadaveric donation. This effect explains 50% of the difference in living donation rates between opt-out and opt-in countries. Countries considering a change in procurement regulations for post-mortem organ donation should therefore anticipate a crowding-out effect. Although only living donations are affected, the crowding-out effect remains relevant because kidneys account for 62% of all transplants.

This effect results from dynamics on both the supply and demand sides. The literature on waiting lists (e.g [30]). highlights both supply-side effects – where an increased availability of cadaveric organs reduces demand for living donations – and demand-side effects – where an increased availability of cadaveric organs increases demand through doctor prioritisation. As the supply of cadaveric organs increases, the reliance on living donations decreases. Moreover, doctors must balance the health of individual patients with the needs of the wider waiting list. This responsibility often results in lower health thresholds for patients to qualify for the waiting list as the supply of cadaveric organs increases [31]. As a result, the increased availability of cadaveric organs further stimulates demand for such organs. Because intrinsic incentives underlying altruistic behaviour remain unaffected, we do not expect a motivational crowding-out of living donations.

Switzerland will switch from opt-in to opt-out in 2026. Our estimation predicts that this legislative shift will increase cadaveric kidney transplants by 6.9 pmp. We also expect a crowding-out effect on living transplants of 2.5 pmp resulting in a net increase of 4.4 kidney transplants pmp (39 additional kidneys).^14^ Considering the waiting list for kidneys as of 2022 [3], the average waiting time for receiving an organ would decrease from 949 to 865 days^15^, presumably resulting in fewer deaths and better health outcomes for patients. Applying this calculation to all opt-in countries in our sample with a total population of 510 million, the model predicts 2,244 additional kidney donors annually.

Practical policies should integrate both deceased and living donation pathways. As our results show, opt-out legislation increases cadaveric donation rates but reduces living donation rates. Recent evidence by Kessler and Roth [32] further underscores that defaults alone are insufficient: their field experiment reveals that changing the wording of donor registration questions – such as switching from opt-in to yes/no formats – has no measurable effect on donor registration rates. Instead, asking individuals to reconsider their donor status outside traditional settings (e.g. driving license applications) proves more effective. This type of proactive engagement not only increases cadaveric registrations but also encourages living donations. Policymakers should leverage these opportunities to promote living donations, thereby offsetting potential decreases caused by increased cadaveric supply. Public campaigns can highlight the unique benefits of living donations, especially in countries transitioning to presumed consent. Maintaining clear donor registries, transparent waiting list policies, and well-trained doctors will help preserve living donation rates as legal frameworks evolve.

Previous studies on the determinants of organ donation rates across countries highlight cultural factors, suggesting that religious beliefs and national legal frameworks influence the willingness of individuals to donate. The differing effects of the predominant procurement rule on cadaveric and living donation rates challenge the cultural imprint hypothesis.

We use deaths from external causes as an instrument for cadaveric transplant rates. However, only a fraction of cadaveric organs originates from such sources, and this proportion varies by country. Incorporating additional instruments (e.g. the number of transplant centres) could improve predictions of cadaveric organ supply; however, this remains an area for future research.

## Data Availability

Data is available from the corresponding author upon request.

## Appendix

**Table 5:** Estimated effects of cadaveric on living kidney rates by sub-sample

Sampling criteria (sample size)	Estimate	Std. error	t value	*p* value
Consent legislation				
Opt-out (13)	−2.503	4.703	−0.532	0.595
Opt-in (9)	−0.320	0.098	−3.271	0.001
Health expenditures per capita				
Above median (11)	−0.246	0.105	−2.337	0.020
Below median (11)	−0.103	0.229	−0.449	0.654
Cadaveric transplant rates				
Above median (11)	−0.421	0.169	−2.486	0.014
Below median (11)	−0.163	0.237	−0.687	0.493
Living transplant rates				
Above median (11)	−0.207	0.116	−1.787	0.075
Below median (11)	−2.648	8.851	−0.299	0.765
Mortality from external causes				
Above median (11)	−0.308	0.120	−2.566	0.011
Below median (11)	−0.628	0.299	−2.102	0.037
Reimbursement program				
Yes (13)	−0.249	0.090	−2.764	0.006
No (9)	−2.983	7.523	−0.396	0.692
Population				
Above median (11)	−0.233	0.081	−2.891	0.004
Below median (11)	−136.214	9,440.628	−0.014	0.989

Note: This table presents the IV coefficients for the effect of the cadaveric kidney rate on the living kidney rate. The model specification is the same as in Table 4 (3), incorporating country fixed effects, a time trend, and a COVID-19 fixed effect. The sample used varies (see the first column) to illustrate the heterogeneity of the effects

**Table 6:** Placebo treatment test

	Living	Cadaveric	Living
	panel linear	first stage	IV
	(1)	(2)	(3)
Cadaveric kidney rate	−0.070^***^ (0.025)		−0.004 (0.184)
Log per capita health expenditure	−0.119 (0.740)	0.887 (1.441)	−0.210 (0.772)
Mortality from kidney diseases		−0.030^***^ (0.010)	
Year	0.187^***^ (0.035)	0.320^***^ (0.066)	0.168^**^ (0.066)
COVID-19	−2.446^***^ (0.458)	−3.625^***^ (0.863)	−2.194^***^ (0.847)
Observations	462	462	462
*R* ^2^	0.864	0.782	0.862
Adjusted *R*^2^	0.857	0.769	0.854
RMSE	2.507	4.802	2.530

Note: ^*^*p* < 0.1; ^**^*p* < 0.05; ^***^*p* < 0.01. All specifications include country fixed effects with clustered standard errors in parentheses. Column 1 presents the results of the fixed effects regression without controlling for the endogeneity of the cadaveric kidney rate (see Eq. 5). Column 2 shows the first stage regression results, where the cadaveric kidney transplant rate is regressed on mortality from kidney diseases, the time trend, and the COVID-19 fixed effect. Column 3 displays the IV estimation results using the same control variables as in Column 2. We conducted the regressions in *R* with the ivreg package

**Table 7:** Consent models and legislation for organ donation by country

Country	Consent system	Foundational legislation
Australia	Opt-in	Section 26, Human Tissue Act 1982 (amended to 1 July 2025)^a^
Austria	Opt-out	Section 62, Federal Law No. 273 of 1 June 1982^b^
Belgium	Opt-out	Section 10, Law of 13 June 1986 (amended to 21 June 2019)^c^
Canada	Opt-in	Section 3, Uniform Human Tissue Donation Act (14 August 1989, model legislation)^d^
Czech Republic	Opt-out	Section 16, Law No. 285/2002 Coll. of 30 May 2002^e^
Denmark	Opt-in	Section 14, Law No. 402 of 13 June 1990^f^
Estonia	Opt-out	Section 9, Tissues Act of 30 January 2002^g^
Finland	Opt-out	Section 9, Law No. 101 of 2 February 2001 (amended by Act No. 653 of 24 June 2010)^h^
France	Opt-out	Caillavet Law No. 76-1181 of December 1976^i^
Germany	Opt-in	Section 3, Transplantation Act (Removal and Transplantation of Organs Act) of 1997^j^
Hungary	Opt-out	Chapter XI, Act CLIV of 1997 on Health Care^k^
Ireland	Opt-in	Followed UK framework: Human Tissue Act 1961 and 2004^l^
Italy	Opt-out	Section 4, Law No. 297 of 1 April 1999^m^
Latvia	Opt-out	Section 2, Law on the Use of Human Tissues and Organs of 15 December 1992 (amended to 29 October 2020)^n^
Lithuania	Opt-in	Section 5, Law of 19 November 1996 (amended to 12 May 2016)^o^
Netherlands	Opt-in	Article 13, Organ Donation Act of 24 May 1996 (before 1 July 2020)^p^
Netherlands	Opt-out	Article 11, Organ Donation Act of 24 May 1996 (revised from 1 July 2020)^q^
New Zealand	Opt-in	Section 3, Human Tissue Act 1964^r^
Norway	Opt-out	Section 7, Law No. 6 of 9 February 1973^s^
Poland	Opt-out	Law of 26 October 1995, replaced by Law of 1 July 2005^t^
Portugal	Opt-out	Article 10, Law No. 12 of 22 April 1993^u^
Slovakia	Opt-out	Section 47, Act No. 277 of 24 August 1994 (amended by Act No. 576/2004)^v^
Slovenia	Opt-out	Article 15, Law on the Removal and Transplantation of Human Body Parts of 26 January 2000^w^
Spain	Opt-out	Article 5, Law No. 30 of 30 October 1979^x^
Sweden	Opt-out	Section 3, Act on the Determination of Death and Organ Donation of 8 June 1995 (amended to 10 October 2024)^y^
Switzerland	Opt-in	Act of 22 March 1996 (replaced by Law No. 810.21 of 8 October 2004)^z^
United States	Opt-in	Section 2, Uniform Anatomical Gift Act of 1968^n/a^

^a^ https://content.legislation.vic.gov.au/sites/default/files/2025-07/82-9860aa047-authorised.pdf

^b^ https://www.ris.bka.gv.at/Dokumente/BgblPdf/1982_273_0/1982_273_0.pdf

^c^ https://www.efod.eu/wp-content/uploads/2021/11/Legislazione-Belgio-ENG.pdf

^d^ https://ulcc-chlc.ca/ULCC/media/EN-Uniform-Acts/Uniform-Human-Tissue-Donation-Act_1.pdf

^e^ https://www.efod.eu/wp-content/uploads/2021/11/Legislazione-Repubblica-Ceca-ENG.pdf

^f^ https://www.efod.eu/wp-content/uploads/2021/11/Legislazione-Danimarca-ENG-1.pdf

^g^ https://www.riigiteataja.ee/akt/928194

^h^ https://www.finlex.fi/en/legislation/collection/2001/101#OT1_OT1

^i^ https://www.legifrance.gouv.fr/jorf/id/JORFTEXT000000699407

^j^ https://www.gesetze-im-internet.de/tpg/

^k^ https://www.efod.eu/wp-content/uploads/2021/11/Legislazione-Ungheria-ENG.pdf

^l^ https://www.efod.eu/wp-content/uploads/2021/11/Legislazione-Irlanda-ENG.pdf

^m^ https://www.normattiva.it/uri-res/N2Ls?urn:nir:stato:legge:1999;91

^n^ https://www.efod.eu/wp-content/uploads/2021/11/Legislazione-Lettonia-ENG.pdf

^o^ https://www.efod.eu/wp-content/uploads/2021/11/Legislazione-Lituania-ENG.pdf

^p^ https://wetten.overheid.nl/BWBR0008066/2019-04-02

^q^ https://wetten.overheid.nl/BWBR0008066/2020-07-01

^r^ https://www.legislation.govt.nz/act/public/1964/0019/1.0/whole.html

^s^ https://app.uio.no/ub/ujur/oversatte-lover/data/lov-19730209-006-eng.pdf

^t^ https://www.poltransplant.pl/Download/prawo/Polish_Transplantation_Act_2005.pdf

^u^ https://www.efod.eu/wp-content/uploads/2021/11/Legislazione-Portogallo-ENG.pdf

^v^ https://www.slov-lex.sk/ezbierky/pravne-predpisy/SK/ZZ/1994/277/20020101.html

^w^ https://www.efod.eu/wp-content/uploads/2021/11/Legislazione-Slovenia-ENG.pdf

^x^ https://noticias.juridicas.com/base_datos/Admin/l30-1979.html

^y^ https://www.notisum.se/rnp/document/?id=19950831

^z^ https://www.fedlex.admin.ch/eli/cc/2007/279/en?print=true

^n/a^ https://www.qcc.cuny.edu/socialSciences/ppecorino/DeathandDying_Text/Uniform%20Anatomical%20Gift%20Act.pdf

## Footnotes

1 It remains unclear whether this result would hold if the authors had used a balanced panel and excluded smaller countries such as Hong Kong and Lebanon.

2 Kidneys and livers are the only organs consistently classified as living donations in official statistics. In the US, one lobe of the lung may also be designated for transplant; however, these instances are not systematically tracked in the dataset we use.

3 For example, 98.6% of kidney and liver transplants in the US come from anonymous donors [33].

4 Financial aspects, time cost, and psychological barriers such as procrastination [34] and discomfort with issues of mortality [35].

5 It can be formally shown that, under opt-out legislation, the family will never override the default. Under opt-in, it will not overrule if *f* (*v*) is symmetric or if *c* is not too large and *f *(*v*) is not extremely skewed (see [15])

6 For example, a donated liver may be divided and transplanted into multiple recipients. Additionally, multiple types of organs are typically recovered from a single deceased donor. When we refer to organ donors, we mean the individuals who donate their organs.

7 This statistic is referred to as intra-class correlation (ICC) in the literature. A high ICC score indicates substantial unobserved heterogeneity in repeated measurements of the response, thereby implying that between-subject random effects should be taken into account. A low ICC score indicates limited individual heterogeneity in the repeated measurements, thereby suggesting that a linear mixed model is not required for analysing the longitudinal data [27].

8 See Tables 6 and 7 in the Appendix.

9 Chapter XX of the International Statistical Classification of Diseases and Related Health Problems 10th Revision (ICD-10) [36].

10 Most eligible donors fall within this age range.

11 In principal, the hierarchical panel structure would allow for an ‘internal instrumental’ variable approach. However, in line with Gui et al. [37], we prefer the external instrument approach because we have a valid and strong instrument.

12 Estonia, Latvia, Lithuania, and Slovakia.

13 We consider the Netherlands to be an opt-in country.

14 We use our estimate from Table 4 to calculate the crowding out effect: 6.9 pmp × 0.361 = 2.49 pmp.

15 In 2023, 400 kidneys were transplanted with patients waiting an average of 949 days. We calculate the waiting time by dividing the patients on the waiting list by the yearly transplants: 1042/439 kidneys/year = 2.37 years × 365 days = 865 days.
